# Tumor‐Resident Microbiota‐Based Risk Model Predicts Neoadjuvant Therapy Response of Locally Advanced Esophageal Squamous Cell Carcinoma Patients

**DOI:** 10.1002/advs.202309742

**Published:** 2024-09-13

**Authors:** Hong Wu, Qianshi Liu, Jingpei Li, Xuefeng Leng, Yazhou He, Yiqiang Liu, Xia Zhang, Yujie Ouyang, Yang Liu, Wenhua Liang, Chuan Xu

**Affiliations:** ^1^ Department of Oncology & Cancer Institute Sichuan Academy of Medical Sciences Sichuan Provincial People's Hospital University of Electronic Science and Technology of China Chengdu Sichuan 610072 P. R. China; ^2^ Sichuan Cancer Hospital & Institute Sichuan Cancer Center School of Medicine University of Electronic Science and Technology of China Chengdu Sichuan 610041 P. R. China; ^3^ Jinfeng Laboratory Chongqing 400039 P. R. China; ^4^ Yu‐Yue Pathology Scientific Research Center Chongqing 400039 P. R. China; ^5^ Thoracic Surgery Department The First Affiliated Hospital of Guangzhou Medical University Guangzhou Guangdong 510230 P. R. China; ^6^ Department of Oncology West China School of Public Health and West China Fourth Hospital Sichuan University Chengdu Sichuan 610041 P. R. China; ^7^ Institute of Pathology and Southwest Cancer Center Ministry of Education of China Southwest Hospital Third Military Medical University (Army Medical University) and Key Laboratory of Tumor Immunopathology Chongqing 400038 P. R. China; ^8^ Acupuncture and Massage College Chengdu University of Traditional Chinese Medicine Chengdu Sichuan 610072 P. R. China

**Keywords:** locally advanced esophageal squamous cell carcinoma, neo‐adjuvant therapy, risk prediction model, tumor‐resident microbiota

## Abstract

Few predictive biomarkers exist for identifying patients who may benefit from neoadjuvant therapy (NAT). The intratumoral microbial composition is comprehensively profiled to predict the efficacy and prognosis of patients with esophageal squamous cell carcinoma (ESCC) who underwent NAT and curative esophagectomy. Least Absolute Shrinkage and Selection Operator (LASSO) regression analysis is conducted to screen for the most closely related microbiota and develop a microbiota‐based risk prediction (MRP) model on the genera of *TM7x*, *Sphingobacterium*, and *Prevotella*. The predictive accuracy and prognostic value of the MRP model across multiple centers are validated. The MRP model demonstrates good predictive accuracy for therapeutic responses in the training, validation, and independent validation sets. The MRP model also predicts disease‐free survival (*p* = 0.00074 in the internal validation set and *p* = 0.0017 in the independent validation set) and overall survival (*p* = 0.00023 in the internal validation set and *p* = 0.11 in the independent validation set) of patients. The MRP‐plus model basing on MRP, tumor stage, and tumor size can also predict the patients who can benefit from NAT. In conclusion, the developed MRP and MRP‐plus models may function as promising biomarkers and prognostic indicators accessible at the time of diagnosis.

## Graphical abstract

The microbiota‐based risk prediction (MRP) model and the integrated MRP‐plus model, incorporating MRP, tumor stage, and tumor size, are devised to discern patients suitable for neoadjuvant therapy (NAT) and prognostic outcomes for those with ESCC. Both the MRP and MRP‐plus models can be practical and reliable tools for guiding treatment strategies in ESCC patients.

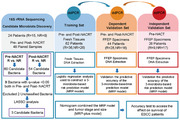

## 1 Introduction

Esophageal carcinoma is one of the most aggressive malignancies worldwide and is associated with a poor 5‐year survival rate of ≈10%.^[^ ^1^ ^]^ There is increasing evidence that preoperative neoadjuvant therapy (NAT) is associated with long‐term survival benefits compared with surgery alone in patients with locally advanced esophageal squamous cell carcinoma (ESCC).^[^ ^2^ ^]^ NAT, which includes neoadjuvant chemotherapy (NACT) and neoadjuvant chemoradiotherapy (NACRT), is the most effective treatment. Definitive NACRT has an excellent complete response (CR) rate of up to 50% in patients with locally advanced esophageal carcinoma.^[^ ^3^ ^]^ The National Comprehensive Cancer Network (NCCN) guidelines indicate that NACT and NACRT before surgery increase the surgical resection rate in ESCC patients. Unfortunately, nearly half of the patients did not benefit from NAT therapy.^[^ ^4^ ^]^ The clinical challenge in such nonresponsive patients is further exacerbated because they usually lose the chance to undergo radical surgery due to disease progression while receiving NAT, resulting in a worse overall prognosis.^[^ ^5^ ^]^

Various types of molecular biomarkers, including genes, microRNAs, DNA methylation, and protein expression, alone or in combination, have been developed to predict the response to cancer therapy and assess survival outcomes in patients with advanced ESCC.^[^ ^6^ ^]^ Although some biomarkers seem useful and valuable, their clinical significance remains unclear or controversial, potentially due to the heterogeneity of patient cohorts, methodologies, and statistical endpoints across studies. Overall, no reliable biomarkers are routinely used in clinical practice to predict the therapeutic response and prognosis of patients with ESCC. Tumor‐resident microbiota has been proven to exist and has become an important hallmark of various cancers.^[^ ^7^ ^]^ Recent studies have shown that microbiota signatures are associated with the progression of ESCC and other cancers.^[^ ^8^ ^]^ Yamamura et al. demonstrated that tumor‐resident *Fusobacterium nucleatum* in esophageal carcinoma tissues was associated with a worse patient prognosis.^[^ ^9^ ^]^ With deeper insight into this field, understanding of the microbiota associated with cancer progression continues to improve rapidly. Thus, there is a need to identify the intra‐tumoral microbiota in these specimens to determine favorable or unfavorable responses to NACRT or NACT, which are robust surrogates for the survival of patients with ESCC.

In this study, we developed a microbiota‐based risk prediction model (MRP model) and an MRP‐plus model (combining tumor stage, tumor size, and MRP model) using 16S rRNA sequencing and droplet digital polymerase chain reaction (ddPCR) testing, validated the feasibility of the risk prediction index and MRP‐plus model for the efficient use of NAT, and predicted the prognosis of patients in clinical cohorts, which could potentially guide therapeutic clinical decision‐making in the treatment of patients with ESCC. In conclusion, we have successfully developed MRP and MRP‐plus models for patients with locally advanced ESCC who received NAT, enabling reliable pretreatment risk prediction for these patients.

## 2 Results

### 2.1 Clinical Characteristics and Microbiota Composition of NAT Responders and Non‐Responders Among ESCC Patients

In total, 180 species from 156 patients with available treatment efficiency data were included in the current analysis. The median age of the patients was 61 years (range, 36–77 years). We performed 16S rRNA sequencing on 48 paired tissue samples (pre‐and post‐NACRT) from 24 patients (Table S1, Supporting Information). The demographic and disease characteristics of the remaining 132 patients, whose tissues were used for ddPCR testing in the training and validation sets, are shown in **Table** **1**.

**Table 1:** Patient demographic and baseline clinical characteristics in the training and validation sets.

	Number of patients		
	Training set	Internal validation set	Independent validation set
Sex			
Male	57	36	22
Female	5	8	4
Age (years)			
≤65	40	31	19
>65	22	13	7
Tumor size (cm)			
≤1.5	26	18	10
>1.5	36	26	16
pTNM			
I	32	31	10
II	9	1	8
III	17	11	6
IV	4	1	2
Tumor location			
Lower	22	17	20
Middle	37	26	5
Upper	3	1	1
Lymph node metastases			
Negative	43	33	15
Positive	19	11	11

**Figure 1:**
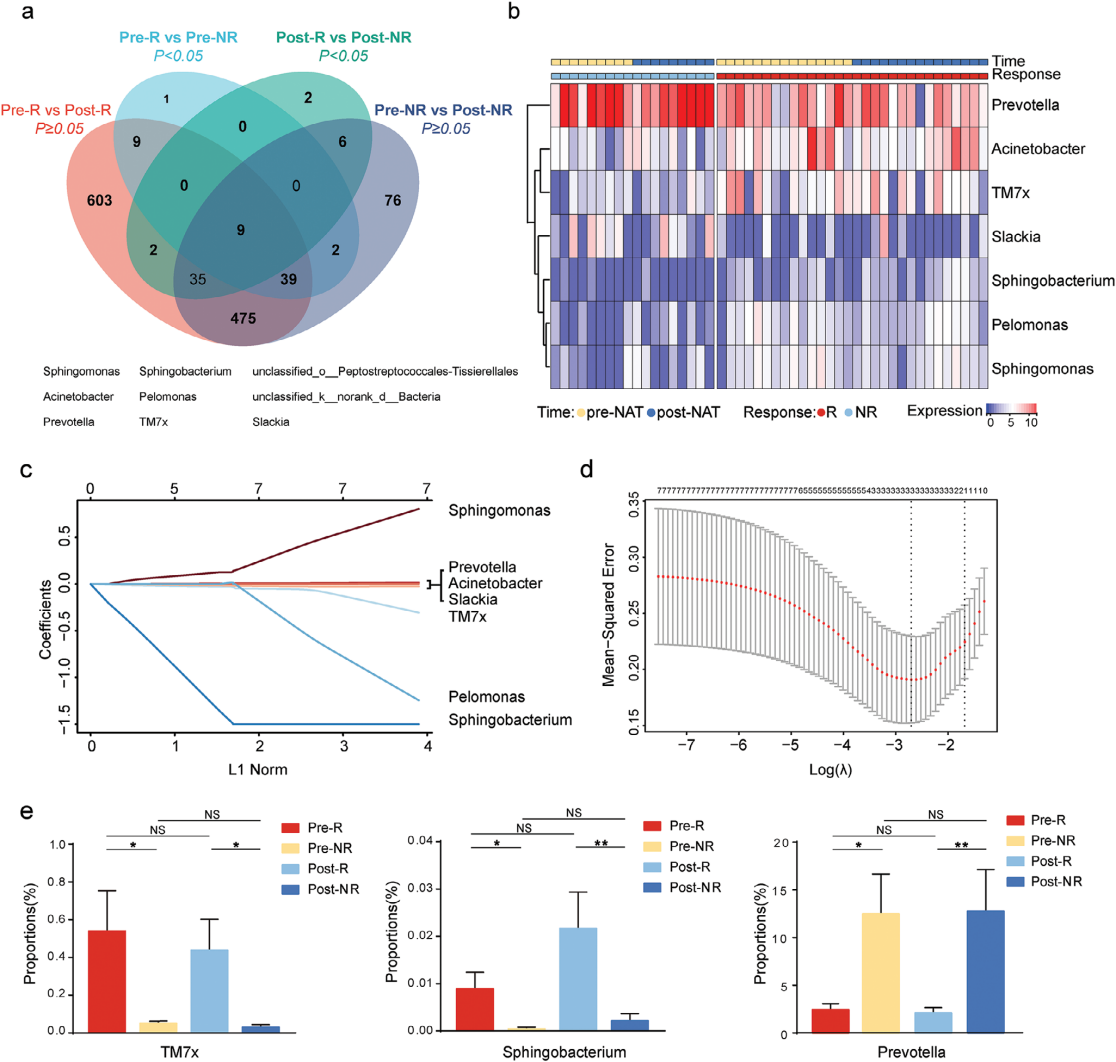
Screening efficiency of the most‐related microbiota for neoadjuvant therapy. a) Venn diagram showing the differential abundance of tumor‐resident microbiota between NACRT responders and non‐responders that remained unchanged between pre‐and post‐NACRT. b) Heatmap analysis of the different tumor‐resident microbiota compositions between NACRT responders(R) and non‐responders (NR) shows no change between pre‐NACRT and post‐NACRT at the genus level. c) LASSO coefficient profiles of the seven differentially expressed bacteria between R and NR. d) The two dotted vertical lines are drawn at the optimal values determined by the minimum criteria (left) and 1−SE criteria (right). e) Fold change of the three LASSO‐selected bacteria. *p < 0.05, **p < 0.01. NS, nonsignificant.

To prevent contamination, we used three environmental background controls. Most microbes in the control samples were *Proteobacteria*, whereas the tissue samples were enriched in *Firmicutes* (Figure S1a, Supporting Information). Unsupervised microbial clustering analysis showed that the environmental background controls and cancer tissues had distinct microbial communities (Figure S1b, Supporting Information). Nine microbiota were screened, with significant differences between responders and non‐responders, no significant changes were observed between pre‐ and post‐NAT treatment (**Figure** **1**a). Seven candidate microbiota have been identified at the genus level (Figure 1b). We conducted Least Absolute Shrinkage and Selection Operator (LASSO) regression analysis and filtered based on the efficiency of the three most closely related microbiota, including *Prevotella, TM7x*, and *Sphingobacterium* (Figure 1c,d). Non‐responders had a higher abundance of *Prevotella*, while responders had higher levels of *TM7x* and *Sphingobacterium* (Figure 1e).

### 2.2 The Microbiota‐Based Risk Prediction Model (MRP) Predicts NAT Resistance in ESCC Patients

**Figure 2:**
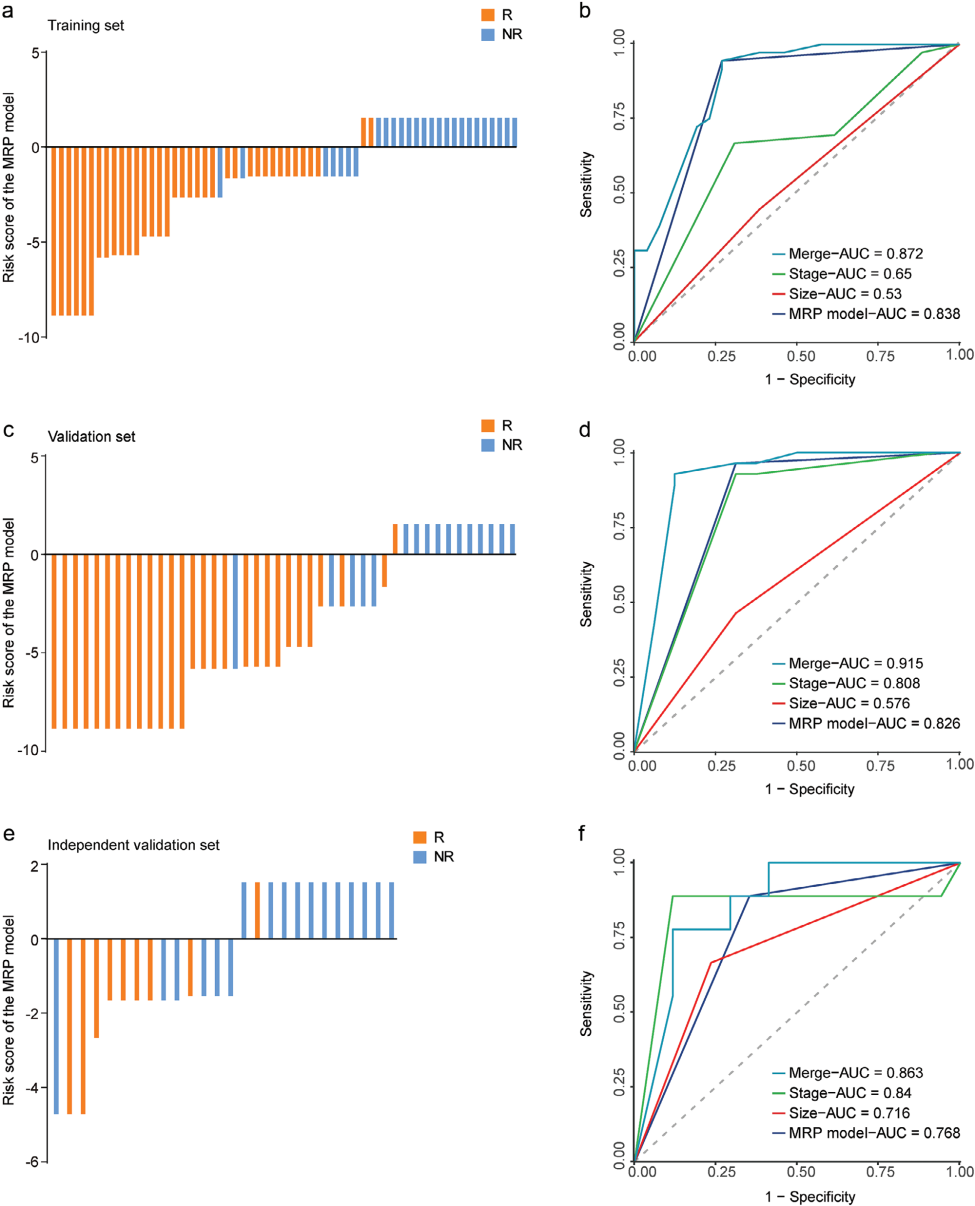
Construction and validation of the microbiota‐based risk prediction model for NAT efficiency. a–f) The MRP model for each patient in different sets. Data are the AUCs of the risk prediction index and clinicopathological features (95% CI). a,b) Training cohort. c,d) Validation set 1. e,f) Independent validation set. AUC = area under the curve.

We examined the presence of tumor‐resident *TM7x, Sphingobacterium*, and *Prevotella* using ddPCR in tumor tissue samples from 132 patients and calculated the optimal cutoff values of each bacterium for treatment efficiency (Figure S2a–c, Supporting Information). We analyzed the prognostic predictive role and correlation with the clinicopathological parameters of each bacterium in two independent research centers (Figure S3a–f and Tables S2–S7, Supporting Information). Using logistic regression analysis, we first developed a risk prediction model based on the Microbiota‐Based Risk Prediction (MRP) model in the clinical training cohort. We calculated the MRP index for each patient based on their abundance levels of the three bacteria, where Logit (*P*) = (4.168616 × status of *Prevotella*) – (3.050354 × status of *TM7x*) – (3.163343 × status of *Sphingobacterium*) – 2.643439 (Table S8, Supporting Information). A decision curve was plotted to compare the benefits of the three filtered bacteria and the MRP model. The net benefit of the MRP model was better than those of *TM7x, Sphingobacterium*, and *Prevotella* for most threshold probabilities in a clinical setting (Figure S2d, Supporting Information). Patients who underwent NAT would benefit from the current MRP model. The risk prediction index predicted reasonable accuracy for NAT efficiency when using the three bacteria (AUC, 0.838; 95% CI, 0.743–0.933), which had better predictive efficiency than tumor size (AUC, 0.53; 95% CI, 0.404–0.656) and tumor stage (AUC, 0.65; 95% CI, 0.52–0.78). The clinicopathological features combined with the MRP model resulted in an even higher predictive power than the MRP model alone (AUC, 0.872; 95% CI, 0.78–0.964; **Figure** **2**a,b). We performed validation analyses in dependent and independent cohorts to assess the applicability of our resistance prediction index to various patients and treatment options. Our MRP model yielded remarkable accuracy in distinguishing non‐responders from responders to NAT in the dependent validation set (AUC, 0.826; 95% CI, 0.704–0.948; Figure 2c,d) and in an independent validation set (AUC, 0.768; 95% CI, 0.608–0.928; Figure 2e,f). Collectively, we successfully validated the ability of our MRP model to predict robust responses to NAT in patients with ESCC.

### 2.3 The Microbiota‐Based Risk Prediction Model Predicts the Survival of Patients with ESCC

To evaluate the clinical applicability of our MRP model, we examined the correlation between the MRP model and the disease‐free survival (DFS) and the overall survival (OS) of patients with ESCC. Patients were classified into high‐ and low‐risk groups based on the MRP model. Low‐risk status was denoted as 0, and high‐risk status was denoted as 1 in the survival analysis. We performed univariate analyses that included age, TNM stage, tumor location, pathological response, tumor size, lymph node metastases, and the MRP model as variables. Among them, the pathological response (HR, 3.45, 95% CI, 1.30–9.21; *p* = 0.013) and the MRP model (HR, 5.01; 95% CI, 1.94–12.98; *p* < 0.001) were identified as significant predictors for OS (**Table** **2**). After adjusting for clinical variables through multivariate Cox regression analysis, the MRP model remained a clinically and statistically significant prognostic index for the prediction of OS (HR, 4.45; 95% CI, 1.26–15.75, *p* = 0.021) (Table 2).

**Table 2:** Univariate and multivariate Cox regression analysis of overall survival.

Variable		Univariable		Multivariable	
		HR^a)^	*p* value	HR	*p* value
Age (years)	≤65	1			
	>65	1.29 (0.48–3.44)	*p* = 0.611		
Tumor stage	I and II	1		1	
	III and IV	1.9 (0.74–4.90)	*p* = 0.185	1.18 (0.43–3.23)	*p* = 0.751
Tumor location	Upper	1			
	Middle	0.51 (0.06–4.19)	*p* = 0.534		
	Lower	1.29 (0.16–10.09)	*p* = 0.809		
Pathologic response	R	1		1	
	NR	3.45 (1.30–9.21)	*p* = 0.013	1.22 (0.32–4.66)	*p* = 0.772
Tumor size (cm)	≤1.5	1		1	
	>1.5	2.01 (0.72–5.63)	*p* = 0.185	2.16 (0.73–6.33)	*p* = 0.162
Lymph node metastases	Negative	1			
	Positive	0.89 (0.29–2.69)	*p* = 0.830		
MRP model^b)^	Low risk	1		1	
	High risk	5.01 (1.94–12.98)	*p* < 0.001	4.45 (1.26–15.75)	*p* = 0.021

^a)^ HR, Hazard Ratio;

^b)^ MRP model, the microbiota‐based risk prediction model.

Moreover, tumor stage (HR, 2.03; 95% CI, 0.92–4.47; *p* = 0.08), pathological response (HR, 2.32, 95% CI, 1.07–5.06; *p* = 0.034), and the MRP model (HR, 3.49; 95% CI, 1.61–7.57; *p* = 0.002) were identified as significant predictors for DFS (**Table** **3**). In the multivariate Cox regression analysis, the MRP model remained a clinically and statistically significant prognostic index for predicting DFS (HR, 3.05; 95% CI, 1.13–8.23, *p* = 0.028) (Table 3).

**Table 3:** Univariate and multivariate Cox regression analysis of disease‐free survival.

Variable		Univariable		Multivariable	
		HR^a)^	*p* value	HR	*p* value
Age (years)	≤65	1			
	>65	1.37 (0.61–3.09)	*p* = 0.441		
Tumor stage	I and II	1		1	
	III and IV	2.03 (0.92–4.47)	*p* = 0.08	1.44 (0.62–3.35)	*p* = 0.402
Tumor location	Upper	1			
	Middle	1.07 (0.14–8.22)	*p* = 0.945		
	Lower	1.66 (0.22–12.77)	*p* = 0.945		
Pathologic response	R	1		1	
	NR	2.32 (1.07–5.06)	*p* = 0.034	1.07 (0.38–2.96)	*p* = 0.901
Tumor size (cm)	≤1.5	1			
	>1.5	1.43 (0.64–3.21)	*p* = 0.385		
Lymph node metastases	Negative	1			
	Positive	1.03 (0.42–2.58)	*p* = 0.941		
MRP model^b)^	Low risk	1		1	
	High risk	3.49 (1.61–7.57)	*p* = 0.002	3.05 (1.13–8.23)	*p* = 0.028

^a)^ HR, Hazard Ratio;

^b)^ MRP model, the microbiota‐based risk prediction model.

**Figure 3:**
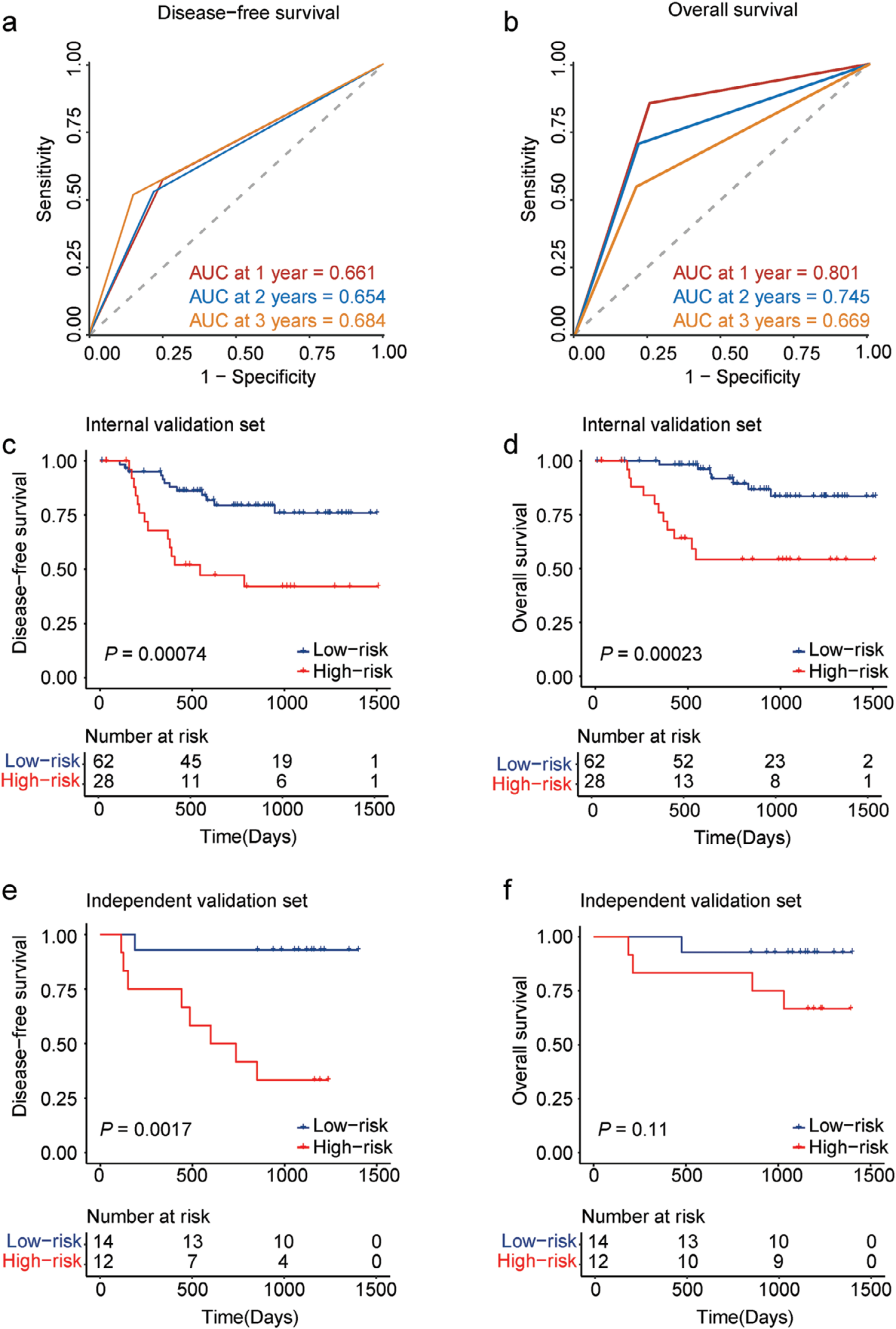
Survival analysis of the microbiota‐based risk prediction model in patients with ESCC. a) Time‐dependent ROC curves and AUCs of the MRP model are used to assess the disease‐free survival of ESCC patients. b) Time‐dependent ROC curves and AUCs are used to assess the overall survival of the MRP model. c) Disease‐free survival curves of low‐ and high‐risk patients as defined by the MRP model. d) Overall survival curves of low‐ and high‐risk patients as defined by the MRP model. e) Disease‐free survival curves of low‐ and high‐risk patients in the independent set. f) Overall survival curves of low‐ and high‐risk patients in the independent set. The *p* values are calculated by log‐rank test for survival.

A time‐dependent ROC curve was used to describe the predictive value of the MRP model. The AUC of the MRP model was 0.661 (95% CI, 0.521–0.8) for one year, 0.654 (95% CI, 0.535–0.774) for two years, and 0.684 (95% CI, 0.558–0.81) for three years (**Figure** **3**a). Regardless of pathological response or non‐response, patients in the high‐risk group had a shorter DFS (HR, 3.463; 95% CI, 1.429–8.394; *p* = 0.00074) than that in the low‐risk group (Figure 3c).

Next, we tested the predictive role of the MRP model in predicting OS. The MRP model was identified as a significant predictor of OS (AUC, 0.801; 95% CI, 0.66–0.942 for one year; AUC, 0.745; 95% CI, 0.612–0.877 for two years; and AUC, 0.669; 95% CI, 0.525–0.813 for three years) (Figure 3b). The patients in the high‐risk group had a shorter OS than those in the low‐risk group (HR, 4.952; 95% CI, 1.681–14.59; *p* = 0.00023) (Figure 3d). An independent validation dataset from the First Affiliated Hospital of Guangzhou Medical University yielded similar results. Patients in the high‐risk group had a shorter DFS (HR, 12.91; 95% CI, 3.366–49.52; *p* = 0.0017) (Figure 3e) but no significant difference in OS (HR, 4.903; 95% CI, 0.842–28.56; *p* = 0.11) (Figure 3f). We have successfully validated that our newly established MRP model can robustly predict the prognosis of patients with locally advanced ESCC.

### 2.4 Construction of Integrated Models to Optimize the Overall Survival Outcome Prediction of ESCC Patients

**Figure 4:**
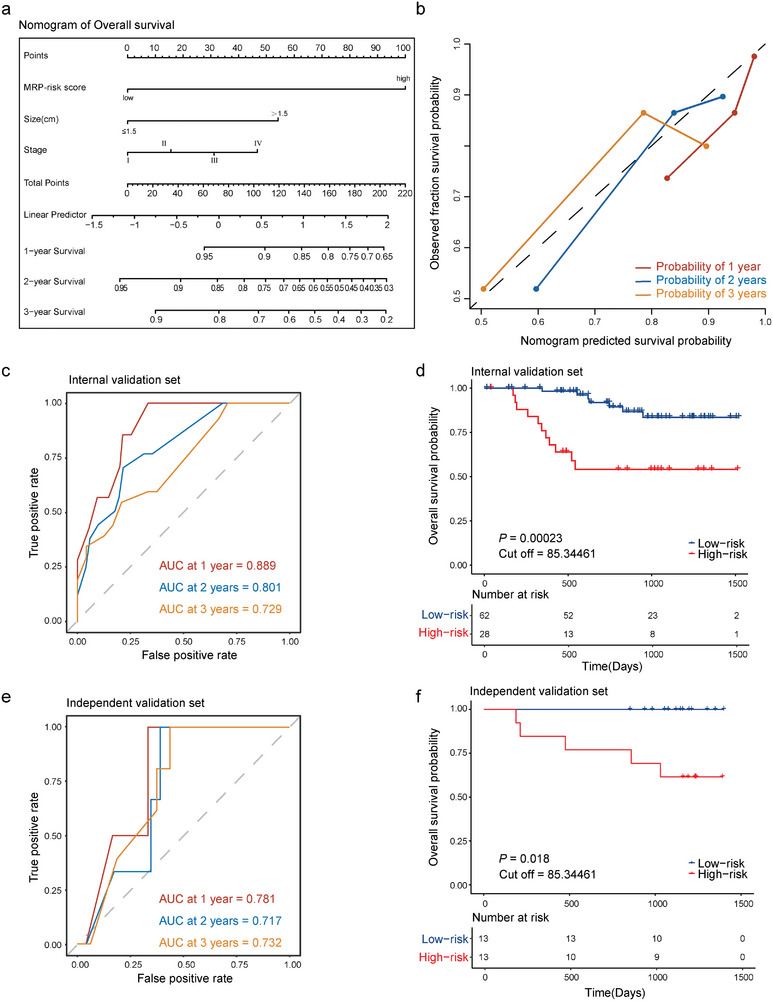
Validation of a nomogram that combines the microbiota‐based risk prediction model and tumor stage and size to predict the survival of patients with ESCC. a) Nomogram for predicting the overall survival of patients after surgery. b) Plots depict the calibration of the model in terms of the agreement between the predicted and observed 3‐year outcomes. Model performance is shown by the plot relative to the 45‐degree line, which represents perfect prediction. c) Time‐dependent ROC curves of the nomogram for survival probability. d) The nomogram illustrates the survival curves of low‐risk and high‐risk patients. e) Time‐dependent ROC curves of the nomogram for the survival probability of the independent set. f) Overall survival curves of low‐risk and high‐risk patients as defined by the nomogram of the independent set. The *p* values are calculated by log‐rank test for survival.

To provide clinicians with a quantitative method for predicting the OS of patients with ESCC who underwent NAT and curative esophagectomy, we constructed nomogram that integrated the MRP model and two clinicopathological risk factors, namely, tumor stage and tumor size (MRP‐plus model) (**Figure** **4**a). The nomogram was developed using data from 90 patients in the training and dependent validation sets, and the calibration curve of the nomogram demonstrated good agreement between prediction and observation (Figure 4b). Time‐dependent ROC analysis at varying follow‐up times showed that the AUCs for OS from one year to three years were 0.889 (95% CI, 0.797–0.98), 0.801 (95% CI, 0.681–0.92), and 0.729 (95% CI, 0.579–0.88) (Figure 4c). The high‐risk group in the MRP‐plus model demonstrated significantly worse OS than that of the low‐risk group (HR, 4.952; 95% CI, 1.681–14.59; *p* = 0.00023) (Figure 4d). In the independent validation set, the time‐dependent ROC analysis at varying follow‐up times showed that the AUC for OS at one year was 0.781 (95% CI, 0.572–0.99), at two years was 0.717 (95% CI, 0.507–0.928), and at three years was 0.732 (95% CI, 0.527–0.938) (Figure 4e). The high‐risk group in the MRP‐plus model exhibited a significantly worse OS than the low‐risk group (*p* = 0.018) (Figure 4f). Thus, the MRP model adds prognostic value to clinicopathological features in predicting the overall survival outcomes of patients with ESCC.

## 3 Discussion

Accumulated evidences demonstrated that preoperative NAT, including NACRT and NACT, can improve survival outcomes in patients with ESCC.^[^ ^10^ ^]^ NAT is the standard treatment for patients with advanced ESCC.^[^ ^11^ ^]^ However, nearly half of the patients did not benefit from these therapies as they suffered from toxicity and missed the opportunity for a curative operation. Therefore, identifying patients who could benefit from such therapies is of great clinical significance and will allow the development of personalized treatment strategies to maximize the therapeutic efficacy for each patient.

Molecular biology technologies have facilitated the rapid advancement of precision medicine for cancer, enabling the implementation of novel multimodal treatment strategies for patients with cancer.^[^ ^12^ ^]^ However, none of these has been utilized in clinical practice. Remarkable progress has recently been made in the detection of bacteria and their use in predicting the efficacy of immunotherapy in cancer patients.^[^ ^13^ ^]^ In this retrospective analysis, we performed a systematic series of experiments to delineate a microbiota‐based risk prediction (MRP) model, including *Prevotella*, *Sphingobacterium*, and *TM7x*, to complement the existing system for predicting the treatment efficiency of NAT and the prognosis of patients with ESCC. Previous studies have reported a significant decrease in *Prevotella* content in the saliva of patients with esophageal cancer compared with healthy individuals, and the ratio of *Porphyromonas* (Pg) to *Prevotella* (Pre) can effectively predict early esophageal cancer occurrence.^[^ ^14^ ^]^ Another study showed a significant increase in the abundance of *Saccharimonadaceae‐TM7x* in cancer‐adjacent tissues, particularly in patients with T4‐stage tumors.^[^ ^15^ ^]^ Reports indicate a higher *Sphingobacterium* content in the urine of patients with bladder cancer.^[^ ^16^ ^]^ Given the therapeutic potential of these findings, as well as their possible applications in cosmetics or as drug carriers in pharmaceutical products, a new biosurfactant produced by *Sphingobacterium* detergens was studied for its hemolytic, cytotoxic, and antiproliferative effects in various cell lines.^[^ ^17^ ^]^

Both NACRT and NACT could improve overall survival rate in locally advanced ESCC patients. Thus, it is expected that MRP and the MRP‐plus models could have a similar predictive value in NACT. The results indicated that these models were equally effective in NACT. However, we did not delve into the underlying mechanisms through which the MRP and MRP‐plus models affect outcomes in both treatment modalities. It may be closely related to the cornerstone role of chemotherapeutic agents in neoadjuvant therapy (NAT). The underlying mechanisms by which this model operates need further exploration.

Moreover, our model could be used for both Formalin‐Fixed Paraffin‐Embedded (FFPE) and fresh tissues before and post‐NAT treatment, thereby increasing its applicability. Clinicians and surgeons can collect tumor tissue specimens using esophagoscopy to clarify the pathological diagnosis before initiating any cancer treatment, which obviates the need for additional biospecimen collection and facilitates the clinical implementation of bacterial detection by ddPCR. There are several different methods for quantifying the microbiota, each with its own strengths and weaknesses. We used ddPCR, which increased sample throughput and decreased the standard deviation.^[^ ^18^ ^]^

Our study has several limitations. First, microbiota selection was based on 16S rRNA sequencing from a single institution, which could potentially introduce inadvertent biases in patient selection and treatment approaches. Second, the microbiota was selected by comparing NAT responders and non‐responders, with no significant change in abundance pre‐ and post‐NAT. This may miss some microbiota that could function well in predicting treatment efficiency but have been changed by NAT. Third, this was a retrospective cohort study with a relatively small number of patients. The results should be validated in future large‐scale prospective clinical trials. Finally, the treatment strategies for patients with ESCC in the multicenter cohorts were different. Although our final model exhibited robust and accurate predictive potential in patients who received different treatment options, the results need to be validated in well‐designed clinical trials with larger patient cohorts to further ensure its predictive ability.

## 4 Conclusion

In conclusion, we have identified and developed the MRP model as a robust and precise predictive tool for resistance to NAT and the survival of patients with locally advanced ESCC. The MRP‐plus model, which combines the MRP model and tumor stage as well as tumor size, was developed as a nomogram. It demonstrates accurate predictive efficiency in identifying patients who could benefit from NAT and the prognosis of patients with ESCC who received NAT and tumor resection. The MRP and MRP‐plus models could help clinicians and surgeons select patients who would benefit from NAT and guide treatment decisions based on differing recurrence risks to provide the best treatment strategy for patients with ESCC.

## 5 Experimental Section

### Study Design and Participants

This study was approved by the Ethics Committee of Sichuan Cancer Hospital and Institute, Sichuan Cancer Centre, School of Medicine, University of Electronic Science and Technology of China (No. SCCHEC‐04‐2022‐004), and the First Affiliated Hospital of Guangzhou Medical University (No. kls2020). Patients whose tissue specimens were available at the Sichuan Cancer Hospital of UESTC and the First Affiliated Hospital of Guangzhou Medical University between May 1, 2018, and September 22, 2022. We performed 16S rRNA sequencing on 48 paired ESCC tumor tissues from 24 patients. Paired tumor tissues were obtained from the same patient before and after NACRT treatment. A significance analysis of 16S rRNA with a false discovery rate of <0.05 was used, to identify the bacteria that were differentially expressed between NAT responders and non‐responders. Based on the 16S rRNA sequencing results and LASSO regression analysis, the presence of three bacteria (*Prevotella*, *Sphingobacterium*, and *TM7x*) using ddPCR was examined to analyze samples from 132 patients who received NACRT (n = 106) and NACT (n = 26). The efficiency and prognostic predictive accuracy of the microbiota‐based risk prediction index (MRP model) and the prognostic value of the MRP‐plus model in different cohorts were investigated. Among the 106 patients who received NACRT, 8 patients died within 90 days after surgery, and 8 could not be contacted which were excluded from the prognosis analysis according to the inclusion and exclusion criteria. The predictive roles of the MRP and MRP‐plus models on patient prognosis and disease‐free survival time in 24 patients receiving NACT treatment were validated. All samples were collected and analyzed after obtaining informed consent from all patients. This study follows the Transparent Reporting of a Multivariable Prediction Model for Individual Prognosis or Diagnosis (TRIPOD) reporting guidelines. Human tissues were collected from the sterile surgery ward and processed in clean, sterile tubes using autoclaved dissection tools.

### Baseline Procedure and Data Collection

Baseline demographic, clinical, and pathological characteristics were recorded, including age, pathological stage, tumor size before surgery, type of NAT, tumor location, presence of lymphatic metastasis, and pathological response scores. Patients with locally advanced esophageal cancer with available clinicopathological characteristics and follow‐up information, were included. NACRT involves at least two cycles of platinum combined with paclitaxel chemotherapy and radiotherapy (at least 40 Gy for GTV). NACT includes at least two cycles of platinum combined with paclitaxel chemotherapy. All patients underwent curative esophagectomy and lymphadenectomy within 3–5 weeks of completing NAT. The exclusion criteria were patients who underwent non‐standard NAT, including those who received only one cycle of chemotherapy. The first observation was either favorable or unfavorable for NAT. The pathologic response was assessed by an independent pathologist using the current tumor regression grading system by examining surgically resected primary tumor specimens following the College of American Pathologists (CAP) scoring system. The pathological response was classified as either favorable (score = 0 [complete response] or 1 [marked response]) or unfavorable (score = 2 [partial response] or 3 [poor or no response]).^[^ ^19^ ^]^ The second observation was the DFS and OS of patients. DFS was defined as the duration of survival from the time of surgery to the date of recurrence or cancer‐related death, whichever occurred first. OS was defined as the time from diagnosis to the date of death or last follow‐up. For survival analyses, patients who could not be contacted or died within 90 days after surgery were excluded.

### DNA Extraction and Droplet Digital PCR

DNA was extracted from all human samples for bacterial quantification and 16S rRNA library preparation. Animal Tissues/Cells Genomic DNA Extraction Kit (Solarbio, Cat. # D1700) for bacterial DNA extraction from fresh tumor tissues and the TIANquick FFPE DNA kit (TIANGEN, DP. #330‐02) for bacterial DNA extraction from FFPE samples, following the manufacturer's instructions.

The DNA previously obtained from ESCC samples was amplified using the EvaGreen ddPCR‐based method. Briefly, for EvaGreen ddPCR, the reaction mix was prepared using 11 µl of 2X QX200 ddPCR EvaGreen Supermix (Cat. No. 1864034; Bio‐Rad Laboratories, Inc.), 0.385 µl of 10 µM Fwd/Rev primer mix, 5.615 µl of RNase‐ and DNase‐free‐water, and 50 ng of cDNA to obtain a final volume of 22 µl. An automated QX200 droplet generator (Bio‐Rad Laboratories, Inc.) was used to generate the droplets. After generation, the droplets were transferred into a 96‐well plate, sealed, and amplified in a C1000 Thermal Cycler (Bio‐Rad Laboratories, Inc.) under the following thermal conditions: 95 °C for 5 min, 40 cycles of amplification at 95 °C for 30 s (denaturation), and 60 °C for 1 min (annealing), droplets stabilization at 4 °C for 5 min and 90 °C for 5 min followed by an infinite hold at 4 °C. A ramp rate of 2 °C s^−1^ was used for amplification. The droplets were read with a QX200 Droplet Reader and analyzed using QuantaSoft software version 1.7.4 (Bio‐Rad). A no‐template control (NTC) for each primer in each detection plate was also included. This involved using water without adding nucleic acid as a substitute for nucleic acids in the reaction. In this study, ddPCR utilized the EvaGreen nucleic acid‐intercalating dye system, and a temperature gradient was employed to screen for the optimal annealing temperature. This helped to identify the ideal annealing/extension temperature, which was beneficial for reducing false positives and false negatives. The threshold between positive and negative droplet populations was set manually using per‐plate no‐template controls as a guide. Microbiota with a higher expression than the cutoff value of 1 and a low expression of 0 were denoted.

### Dealing with Contaminations in Bacterial 16S rRNA Profiling of Low Microbial Biomass Samples

The tissue‐resident microbiota has a much lower bacterial load by several orders of magnitude than the gut microbiome and a significantly higher host genome contamination. Quantifying the absolute abundance and profiles of microbial communities is challenging. The entire experimental procedure was carefully optimized from beginning to end to accurately and sensitively detect and profile the tissue‐resident microbiota.^[^ ^20^ ^]^ DMEM/H was introduced which underwent all the sample preparation procedures, including exposure to the operating room environment, laboratory environment, and tumor tissue shredding process as an environmental background control (EBC). Additionally, DEPC‐treated water was used as a no‐template control (NTC) to account for the various sources of contamination from the hospital and laboratory environments and the different stages of handling and processing of the samples. The 16S rRNA seq raw data of control was uploaded and tissue samples on https://www.ncbi.nlm.nih.gov/sra.

### Isolating the Target Bacteria and Design Specific Primers

Various intra‐tumor microorganisms through tissue culture were isolated and enriched in the preliminary study.^[^ ^20^ ^]^ To isolate anaerobic or aerobic bacteria, ESCC tissue samples (≈0.1 g) were minced into small pieces and homogenized with a glass homogenizer in 1 mL ice‐cold DMEM High Glucose (DMEM/H; HyClone) under sterile conditions. For aerobic culture, 100 mL sample homogenate was plated on Columbia blood agar (CBA; JX601) supplemented with 5% sheep blood (Solarbio, catalog no. TX0030) aerobically with 5% CO_2_. For anaerobic culture, 100 mL sample homogenate was plated on CBA (JX601) supplemented with 5% sheep blood (Solarbio, catalog no. TX0030) and placed in an anaerobic bag (AnaeroPouch). The plates were incubated at 37 °C for 3 days under aerobic or anaerobic conditions (Figure S1c,d, Supporting Information). Bacterial colonies were collected on day 3. The collected colonies were prepared for 16S rRNA sequencing (Figure S1e, Supporting Information). Based on the sequencing results, multiple primers targeting the genus were designed. Then, qRT‐PCR was performed on the enriched intra‐tumor bacteria to confirm the specificity of primers (Figure S1f, Supporting Information).

### 16S rRNA Amplification and Sequencing

For microbiota library construction, the V3‐V4 hypervariable region of the 16S gene was amplified from samples according to a previously described method with some optimizations. Media was used that underwent all sample preparation procedures as environmental negative control and DEPC‐treated water as a no‐template control. The V3‐V4 hypervariable region of the 16S gene was amplified using the ABI GeneAmp 970. The PCR amplification products were separated by 2% gel electrophoresis and purified using the AxyPrep DNA Gel Extraction Kit (Axygen Biosciences) following the manufacturer's instructions. Finally, the PE300 library was established using amplification procedures following the standard protocol of Illumina MiSeq (Illumina, San Diego, CA, USA). The samples were sequenced on an Illumina MiSeq PE300 platform (Shanghai Meiji Biopharma Technology Co., Ltd.). To minimize the effects of sequencing depth on alpha and beta diversity measures, the number of sequences from each sample was reduced to 20 000, yielding an average Good's coverage of 97.90%.

### Statistical Analyses

Statistical analyses were performed using GraphPad Prism (RRID: SCR_002798) version 9.2.0 (GraphPad Software). The baseline characteristics of patients were compared between groups using χ^2^ tests. The LASSO logistic regression model was used for treatment efficiency analysis based on 16S rRNA sequencing data. A microbiota‐based risk prediction (MRP) model using ddPCR results was developed and logistic regression analyses. Kaplan‐Meier analysis was used to estimate the correlations between variables and DFS and OS, and the log‐rank test was used to compare survival curves. The Cox regression model was applied for multivariable survival analysis and used Cox regression coefficients to generate nomograms. Calibration plots were generated to explore the performance of the nomograms. The x‐axis represents the predictions calculated using the nomogram, and the y‐axis represents the survival of our patients. Nomogram and calibration plots were generated using the “rms” package in R software. Statistical significance was set at *p* < 0.05. Time‐dependent ROC curve analysis was performed using R software version 4.2.3 and the “timeROC” package.

### Ethics Approval and Consent to Participate

All samples were collected and analyzed after informed consent was obtained from the patients.

## Conflict of Interest

The authors declare no conflict of interest.

## Author Contributions

H.W., Q.L., J.L., and X.L. contributed equally to this work. Dr. Xu, Dr. Liu, and Dr. Liang (senior co‐authors) had full access to all of the data in the study. They took responsibility for the data's integrity and the accuracy of the data analyses. C.X., W.L., and H.W. performed conceptualization and design; H.W., Q.L., and X.L. performed the acquisition, analysis, or interpretation of data and experiments; C. X., W.L., and H.W. drafted the manuscript; C.X., W.L., Y.H., Y.O., Q.L., X.Z., and H.W. performed critical revision of the manuscript for important intellectual content; H.W., Q.L., and Y.H. performed the statistical analysis; C.X. acquired funding; J.L., Y.L., and Y.H. provided administrative, technical, or material support; C.X. and W.L. did supervision.

## Supporting information

Supporting Information

## Data Availability

The data that support the findings of this study are available on request from the corresponding author. The data are not publicly available due to privacy or ethical restrictions.
