# Supplementary material for: Tumor‐Resident Microbiota‐Based Risk Model Predicts Neoadjuvant Therapy Response of Locally Advanced Esophageal Squamous Cell Carcinoma Patients
Source: Adv Sci (Weinh). 2024 Sep 13;11(41):2309742. doi: 10.1002/advs.202309742 (PMC11538710; doi:10.1002/advs.202309742)
Supplement: Supplementary file 1 — Supporting Information [file ADVS-11-2309742-s001.docx]

Tumor-Resident Microbiota-Based Risk Model Predicts Neoadjuvant Therapy Response of Locally Advanced Esophageal Squamous Cell Carcinoma Patients

**Authors**

Hong Wu^1,2,4,7^, Qianshi Liu^1,2,4,7^, Jingpei Li^3^, Xuefeng Leng^2^, Yazhou He^5^, Yiqiang Liu^1,4^, Xia Zhang^6^, Yujie Ouyang^8^, Yang Liu^2*^, Wenhua Liang^3*^, Chuan Xu^1,4,7*^


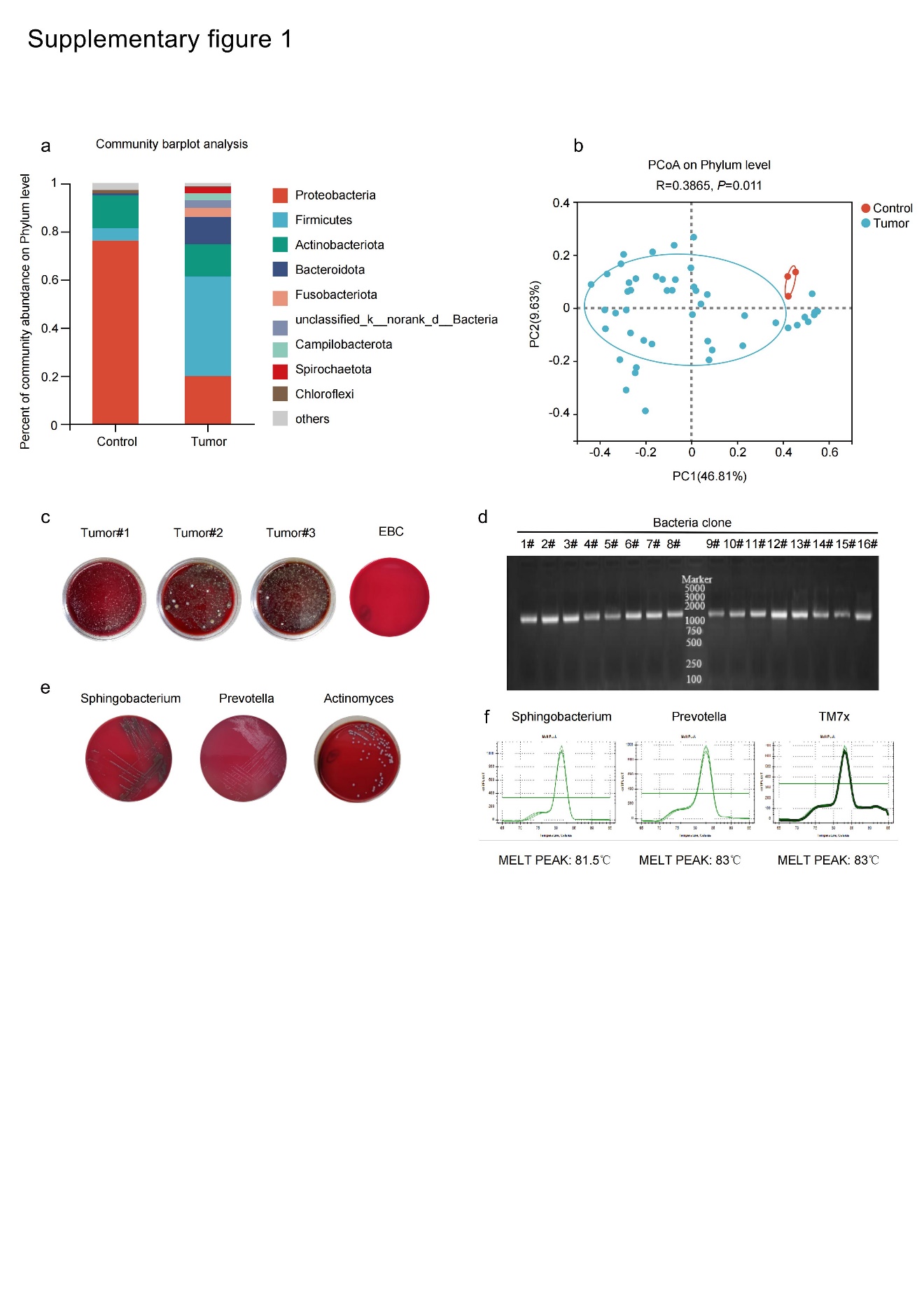


**Figure S1. Environmental controls are compared with tumor tissues to exclude contamination. (a)** Bar plot of the phylogenetic composition of common bacterial taxa (>1% abundance) at the phylum level in environmental control and tumor tissues. **(b)** Unsupervised clustering microbiome analysis of the control and cancer tissues. **(c)** Representative pictures showing bacterial cultures of EBC (environmental background controls) and ESCC tumors on CBA (Columbia blood agar base) plates. **(d)** Bacteria DNA was amplified by PCR assay and amplification products were analyzed by agarose gel electrophoresis. The product length was 1500 bp. **(e)** Bacterial colonies were picked for cloning. **(f)** qRT-PCR verified the specificity of the primers including *Sphingobacterium, Prevotella,* and *TM7x.*


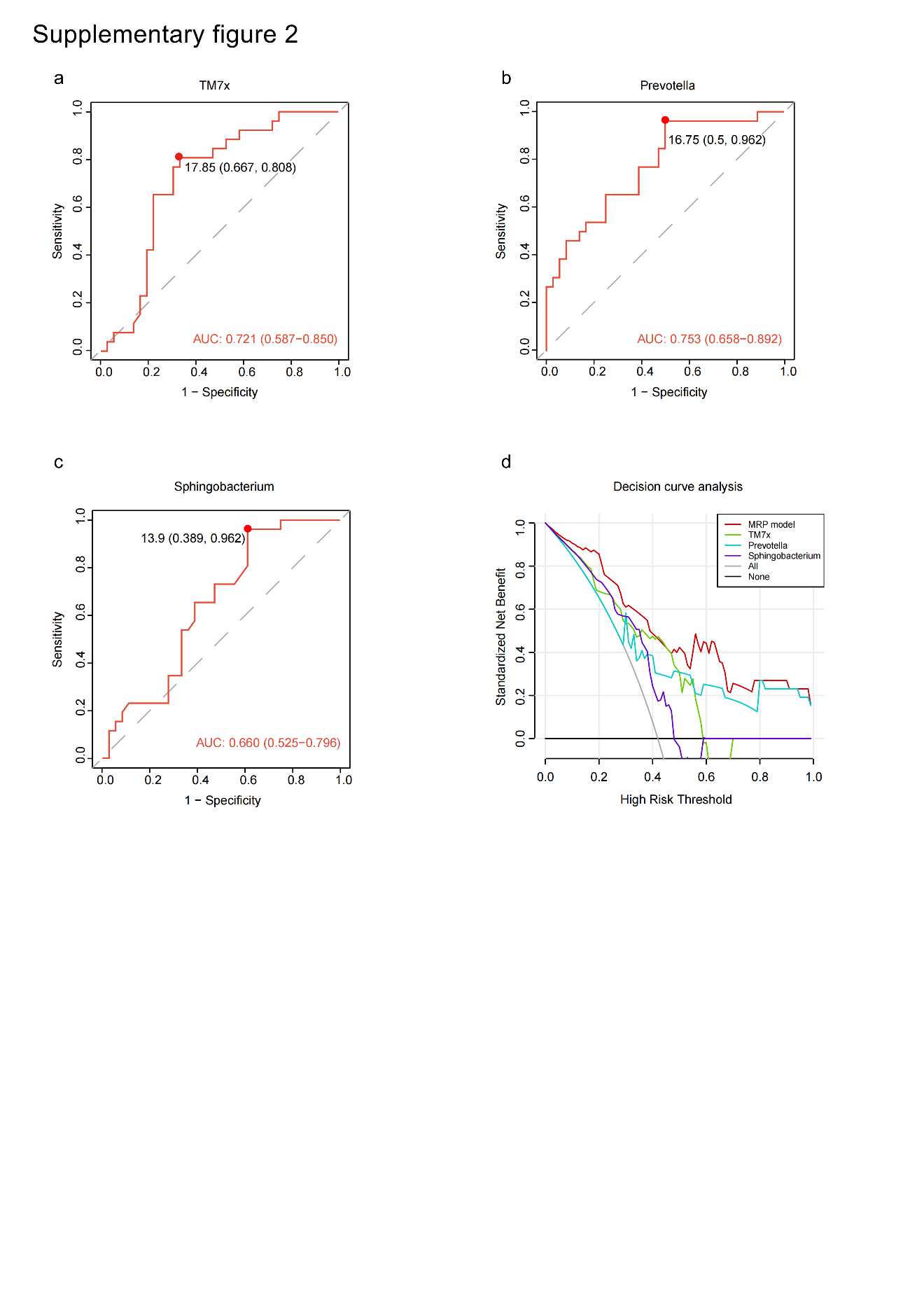


**Figure S2. The cut-off value of each bacterium for NAT response. (a-c)** ROC curves and AUCs of each bacterium are used to assess the response to NAT in patients with ESCC. **(d)** Decision curve analysis of the model based on filtered bacteria.


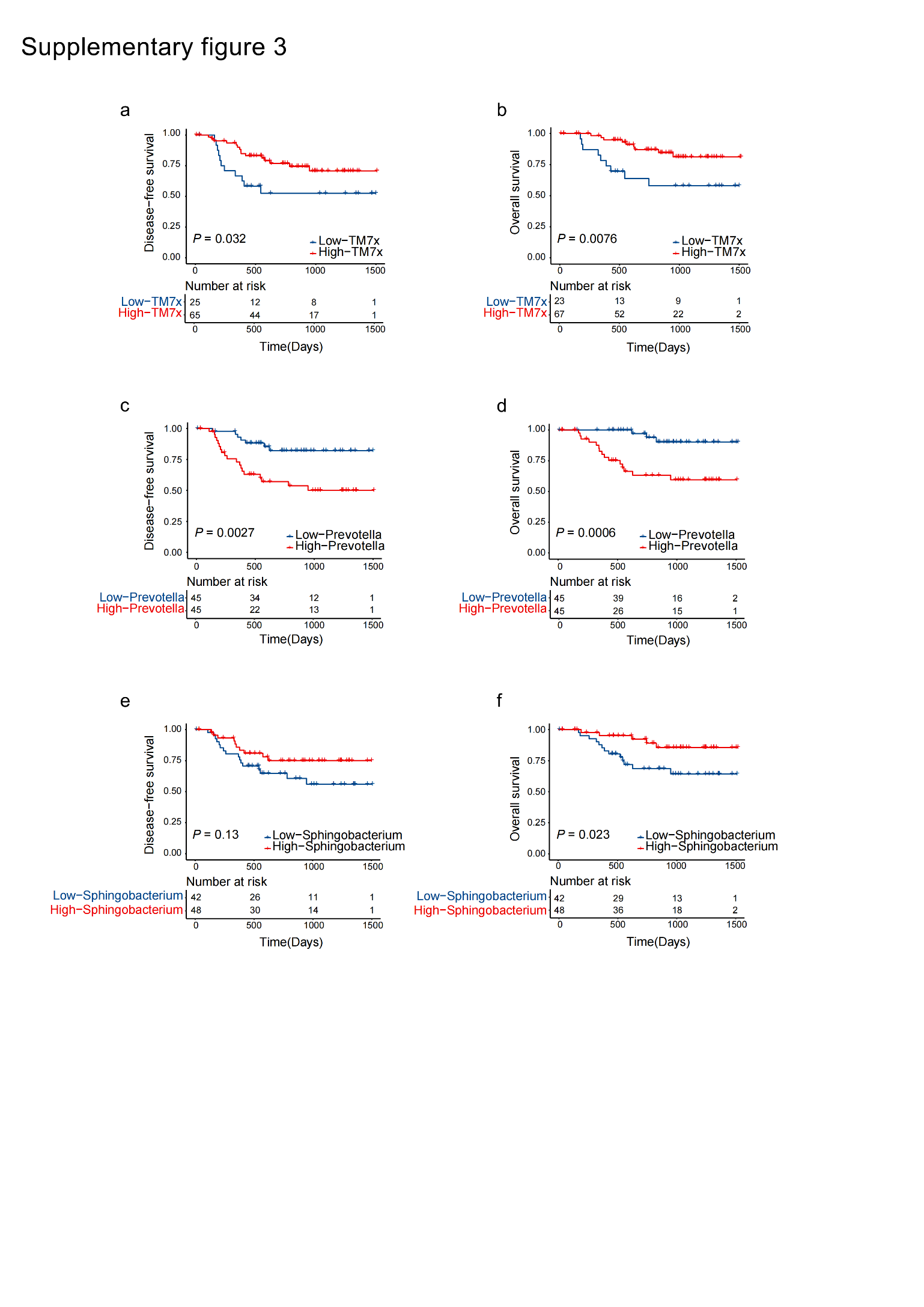


**Figure S3. The role of each bacterium in predicting the disease-free survival and overall survival of ESCC patients who underwent NAT. (a and b)** Role of *TM7x* expression in predicting the disease-free survival and overall survival of patients with ESCC. **(c and d)** The role of *Prevotella* expression in predicting disease-free survival and overall survival in patients with ESCC. **(e and f)** Role of *Sphingobacterium* expression in predicting the disease-free and overall survival of patients with ESCC. The *P* values are calculated by log-rank test for survival.

**Table S1. The Clinicopathological Characteristics of ESCC Patients Tested by 16S rRNA Sequencing.**

| **Sample** | **Sex** | **Age (years)** | **Response** | **ypTNM** |
| --- | --- | --- | --- | --- |
| 1 | Male | 56 | Response | ypT0N0M0 |
| 2 | Male | 57 | Response | ypT0N0M0 |
| 3 | Male | 69 | Response | ypT0N1M0 |
| 4 | Male | 54 | Response | ypT3N1M0 |
| 5 | Male | 58 | Response | ypT0N1M0 |
| 6 | Male | 75 | Response | ypT2N1M0 |
| 7 | Male | 52 | Response | ypTisN0M0 |
| 8 | Male | 56 | Response | ypT1N0M0 |
| 9 | Female | 60 | Response | ypT0N1M0 |
| 10 | Male | 65 | Response | ypT2N0M0 |
| 11 | Male | 53 | Response | ypT0N0M0 |
| 12 | Male | 70 | Response | ypT1N1M0 |
| 13 | Male | 65 | Response | ypT0N0M0 |
| 14 | Female | 69 | Response | ypT0N0M0 |
| 15 | Male | 66 | Response | ypT0N0M0 |
| 16 | Male | 73 | Non-response | ypT3N1M0 |
| 17 | Male | 67 | Non-response | ypT2N1M0 |
| 18 | Male | 53 | Non-response | ypT2N0M0 |
| 19 | Female | 56 | Non-response | ypT2N1M0 |
| 20 | Male | 50 | Non-response | ypT3N2M0 |
| 21 | Male | 60 | Non-response | ypT3N1M0 |
| 22 | Male | 72 | Non-response | ypT2N0M0 |
| 23 | Male | 68 | Non-response | ypT3N1M0 |
| 24 | Male | 63 | Non-response | ypT3N2M0 |

**Table S2. Correlation Analysis Between *Prevotella* Expression and Clinicopathological Parameters of ESCC Patients.**

| **Variable** | **Number of cases** | ***Prevotella*** | | ***P* Value** |
| --- | --- | --- | --- | --- |
|  |  | High expression | Low expression |  |
|  |  | (*N*=59) | (*N*=47) |  |
| **Age (years)** |  |  |  | 1 |
| ≤65 | 71 | 40 | 31 |  |
| >65 | 35 | 19 | 16 |  |
| **Sex** |  |  |  | 0.103 |
| Female | 13 | 4 | 9 |  |
| Male | 93 | 55 | 38 |  |
| **Tumor size (cm)** |  |  |  | 0.6888 |
| ≤1.5 | 44 | 26 | 18 |  |
| >1.5 | 62 | 33 | 29 |  |
| **pTNM** |  |  |  | 0.081 |
| Ⅰ-Ⅱ | 73 | 36 | 37 |  |
| Ⅲ-Ⅳ | 33 | 23 | 10 |  |
| **Lymph node metastases** |  |  |  | 0.3957 |
| Negative | 78 | 41 | 37 |  |
| Positive | 28 | 18 | 10 |  |
| **Pathologic response** |  |  |  | <0.0001 |
| Response | 64 | 23 | 41 |  |
| Non-response | 42 | 36 | 6 |  |
| **Tumor location** |  |  |  | 0.6465 |
| Upper | 4 | 2 | 2 |  |
| Middle | 63 | 33 | 30 |  |
| Lower | 39 | 24 | 15 |  |

**Table S3. Correlation Analysis Between *TM7x* Expression and Clinicopathological Parameters of ESCC Patients.**

| **Variable** | **Number of cases** | ***TM7x*** | | ***P* Value** |
| --- | --- | --- | --- | --- |
|  |  | High expression | Low expression |  |
|  |  | (*N*=49) | (*N*=57) |  |
| **Age (years)** |  |  |  | 1 |
| ≤65 | 71 | 33 | 38 |  |
| >65 | 35 | 16 | 19 |  |
| **Sex** |  |  |  | 1 |
| Female | 13 | 6 | 7 |  |
| Male | 93 | 43 | 50 |  |
| **Tumor size (cm)** |  |  |  | 1 |
| ≤1.5 | 44 | 20 | 24 |  |
| >1.5 | 62 | 29 | 33 |  |
| **pTNM** |  |  |  | 0.2465 |
| Ⅰ-Ⅱ | 73 | 37 | 36 |  |
| Ⅲ-Ⅳ | 33 | 12 | 21 |  |
| **Lymph node metastases** |  |  |  | 0.5236 |
| Negative | 78 | 38 | 40 |  |
| Positive | 28 | 11 | 17 |  |
| **Pathologic response** |  |  |  | <0.0001 |
| Response | 64 | 44 | 20 |  |
| Non-response | 42 | 5 | 37 |  |
| **Tumor location** |  |  |  | 0.7145 |
| Upper | 4 | 2 | 2 |  |
| Middle | 63 | 31 | 32 |  |
| Lower | 39 | 16 | 23 |  |

**Table S4. Correlation Analysis Between *Sphingobacterium* Expression and Clinicopathological Parameters of ESCC Patients.**

| **Variable** | **Number of cases** | ***Sphingobacterium*** | | ***P* Value** |
| --- | --- | --- | --- | --- |
|  |  | High expression | Low expression |  |
|  |  | (*N*=37) | (*N*=69) |  |
| **Age (years)** |  |  |  | 0.7561 |
| ≤65 | 71 | 26 | 45 |  |
| >65 | 35 | 11 | 24 |  |
| **Sex** |  |  |  | 0.55 |
| Female | 13 | 6 | 7 |  |
| Male | 93 | 31 | 62 |  |
| **Tumor size (cm)** |  |  |  | 0.3758 |
| ≤1.5 | 44 | 18 | 26 |  |
| >1.5 | 62 | 19 | 43 |  |
| **pTNM** |  |  |  | 0.3743 |
| Ⅰ-Ⅱ | 73 | 28 | 45 |  |
| Ⅲ-Ⅳ | 33 | 9 | 24 |  |
| **Lymph node metastases** |  |  |  | 0.8994 |
| Negative | 78 | 28 | 50 |  |
| Positive | 28 | 9 | 19 |  |
| **Pathologic response** |  |  |  | <0.0001 |
| Response | 64 | 35 | 29 |  |
| Non-response | 42 | 2 | 40 |  |
| **Tumor location** |  |  |  | 0.1405 |
| Upper | 4 | 2 | 2 |  |
| Middle | 63 | 26 | 37 |  |
| Lower | 39 | 9 | 30 |  |

**Table S5. Correlation Analysis Between *Prevotella* Expression and Clinicopathological Parameters of ESCC Patients.**

| **Variable** | **Number of cases** | ***Prevotella*** | | ***P* Value** |
| --- | --- | --- | --- | --- |
|  |  | High expression | Low expression |  |
|  |  | (*N*=25) | (*N*=1) |  |
| **Age (years)** |  |  |  | 1 |
| ≤65 | 19 | 18 | 1 |  |
| >65 | 7 | 7 | 0 |  |
| **Sex** |  |  |  | 1 |
| Female | 4 | 4 | 0 |  |
| Male | 22 | 21 | 1 |  |
| **Tumor size (cm)** |  |  |  | 1 |
| ≤1.5 | 10 | 10 | 0 |  |
| >1.5 | 16 | 15 | 1 |  |
| **pTNM** |  |  |  | 1 |
| Ⅰ-Ⅱ | 18 | 17 | 1 |  |
| Ⅲ-Ⅳ | 8 | 8 | 0 |  |
| **Lymph node metastases** |  |  |  | 1 |
| Negative | 15 | 14 | 1 |  |
| Positive | 11 | 11 | 0 |  |
| **Pathologic response** |  |  |  | 0.7416 |
| Response | 9 | 8 | 1 |  |
| Non-response | 17 | 17 | 0 |  |
| **Tumor location** |  |  |  | 0.8556 |
| Upper | 1 | 1 | 0 |  |
| Middle | 5 | 5 | 0 |  |
| Lower | 20 | 19 | 1 |  |

**Table S6. Correlation Analysis Between *Sphingobacterium* Expression and Clinicopathological Parameters of ESCC Patients.**

| **Variable** | **Number of cases** | ***Sphingobacterium*** | | ***P* Value** |
| --- | --- | --- | --- | --- |
|  |  | High expression | Low expression |  |
|  |  | (*N*=9) | (*N*=17) |  |
| **Age (years)** |  |  |  | 1 |
| ≤65 | 19 | 7 | 12 |  |
| >65 | 7 | 2 | 5 |  |
| **Sex** |  |  |  | 0.3122 |
| Female | 4 | 0 | 4 |  |
| Male | 22 | 9 | 13 |  |
| **Tumor size (cm)** |  |  |  | 0.3789 |
| ≤1.5 | 10 | 5 | 5 |  |
| >1.5 | 16 | 4 | 12 |  |
| **pTNM** |  |  |  | 0.2569 |
| Ⅰ-Ⅱ | 18 | 8 | 10 |  |
| Ⅲ-Ⅳ | 8 | 1 | 7 |  |
| **Lymph node metastases** |  |  |  | 0.2752 |
| Negative | 15 | 7 | 8 |  |
| Positive | 11 | 2 | 9 |  |
| **Pathologic response** |  |  |  | 0.0388 |
| Response | 9 | 6 | 3 |  |
| Non-response | 17 | 3 | 14 |  |
| **Tumor location** |  |  |  | 0.7428 |
| Upper | 1 | 0 | 1 |  |
| Middle | 5 | 2 | 3 |  |
| Lower | 20 | 7 | 13 |  |

**Table S7. Correlation Analysis Between *TM7x* Expression and Clinicopathological Parameters of ESCC Patients.**

| **Variable** | **Number of cases** | ***TM7X*** | | ***P* Value** |
| --- | --- | --- | --- | --- |
|  |  | High expression | Low expression |  |
|  |  | (*N*=7) | (*N*=19) |  |
| **Age (years)** |  |  |  | 0.1675 |
| ≤65 | 19 | 7 | 12 |  |
| >65 | 7 | 0 | 7 |  |
| **Sex** |  |  |  | 0.6041 |
| Female | 4 | 2 | 2 |  |
| Male | 22 | 5 | 17 |  |
| **Tumor size (cm)** |  |  |  | 0.1004 |
| ≤1.5 | 10 | 5 | 5 |  |
| >1.5 | 16 | 2 | 14 |  |
| **pTNM** |  |  |  | 0.5311 |
| Ⅰ-Ⅱ | 18 | 6 | 12 |  |
| Ⅲ-Ⅳ | 8 | 1 | 7 |  |
| **Lymph node metastases** |  |  |  | 0.6796 |
| Negative | 15 | 5 | 10 |  |
| Positive | 11 | 2 | 9 |  |
| **Pathologic response** |  |  |  | 0.943 |
| Response | 9 | 3 | 6 |  |
| Non-response | 17 | 4 | 13 |  |
| **Tumor location** |  |  |  | 0.7458 |
| Upper | 1 | 0 | 1 |  |
| Middle | 5 | 1 | 4 |  |
| Lower | 20 | 6 | 14 |  |

**Table S8. The Expression of Three Bacteria Tested by ddPCR and MRP Index of Each ESCC Sample.**

| **Variable** | **Age (years)** | | **Sex** | ***TM7x*** | ***Prevotella*** | ***Sphingobacterium*** | **Risk score** |
| --- | --- | --- | --- | --- | --- | --- | --- |
| **Training set** |  | |  |  |  |  |  |
| 1 | 60 | | Male | 13.1 | 102.6 | 0.43 | 1.525177 |
| 2 | 66 | | Male | 0.31 | 16.1 | 9.4 | -2.643439 |
| 3 | 70 | | Male | 0.72 | 14.4 | 3.8 | -2.643439 |
| 4 | 51 | | Famale | 1.3 | 10.1 | 45.5 | -5.806782 |
| 5 | 63 | | Male | 2.1 | 46.4 | 18 | -1.638166 |
| 6 | 62 | | Male | 7.9 | 166.7 | 0.74 | 1.525177 |
| 7 | 52 | | Male | 34.9 | 11.1 | 8 | -5.693793 |
| 8 | 68 | | Male | 18.8 | 115.2 | 0 | -1.525177 |
| 9 | 64 | | Male | 63.8 | 46.3 | 0.74 | -1.525177 |
| 10 | 52 | | Male | 0.84 | 19.8 | 0.12 | 1.525177 |
| 11 | 54 | | Male | 32.9 | 22.7 | 1.09 | -1.525177 |
| 12 | 64 | | Male | 10.2 | 9.9 | 4.2 | -2.643439 |
| 13 | 61 | | Male | 3.9 | 1739 | 20 | -1.638166 |
| 14 | 64 | | Male | 12.1 | 13.9 | 1.7 | -2.643439 |
| 15 | 36 | | Male | 2.1 | 343 | 11.7 | 1.525177 |
| 16 | 62 | | Male | 22.3 | 12.3 | 24.5 | -8.857136 |
| 17 | 62 | | Male | 16.8 | 16.5 | 17.4 | -5.806782 |
| 18 | 49 | | Male | 7.2 | 90.5 | 7.3 | 1.525177 |
| 19 | 71 | | Male | 23 | 129.3 | 67.3 | -4.68852 |
| 20 | 68 | | Male | 32 | 40.3 | 49.2 | -4.68852 |
| 21 | 70 | | Male | 26.2 | 76.1 | 18.8 | -4.68852 |
| 22 | 61 | | Male | 12.2 | 24.8 | 3.1 | 1.525177 |
| 23 | 56 | | Male | 4 | 90.7 | 35.5 | -1.638166 |
| 24 | 60 | | Famale | 33.8 | 250 | 7.1 | -1.525177 |
| 25 | 69 | | Male | 53 | 52.1 | 91.6 | -4.68852 |
| 26 | 66 | | Male | 12.7 | 35.8 | 0.12 | 1.525177 |
| 27 | 49 | | Male | 37 | 398 | 10 | -1.525177 |
| 28 | 63 | | Male | 71.5 | 8.8 | 14.2 | -8.857136 |
| 29 | 66 | | Male | 36.6 | 17 | 0.25 | -1.525177 |
| 30 | 54 | | Male | 8.7 | 1191 | 3.3 | 1.525177 |
| 31 | 66 | | Male | 1.4 | 13.9 | 7.9 | -2.643439 |
| 32 | 52 | | Male | 5.2 | 111.6 | 7.4 | 1.525177 |
| 33 | 49 | | Male | 38.3 | 108.3 | 8.8 | -1.525177 |
| 34 | 62 | | Male | 22.4 | 18.7 | 0.37 | -1.525177 |
| 35 | 64 | | Male | 70 | 1.9 | 43.1 | -8.857136 |
| 36 | 62 | | Male | 30.2 | 4.7 | 24.9 | -8.857136 |
| 37 | 62 | | Male | 19.7 | 11.5 | 38.3 | -8.857136 |
| 38 | 53 | | Male | 4.1 | 3300 | 0.55 | 1.525177 |
| 39 | 50 | | Male | 37.2 | 17.3 | 3.4 | -1.525177 |
| 40 | 72 | | Male | 60.3 | 6.2 | 0.48 | -5.693793 |
| 41 | 70 | | Male | 2.6 | 62.9 | 8.3 | 1.525177 |
| 42 | 71 | | Famale | 43.4 | 2.2 | 0.95 | -5.693793 |
| 43 | 56 | | Male | 30.3 | 363 | 8.5 | -1.525177 |
| 44 | 54 | | Male | 35.6 | 723 | 0.78 | -1.525177 |
| 45 | 66 | | Male | 0.62 | 19.3 | 4.9 | 1.525177 |
| 46 | 51 | | Male | 48.3 | 23.9 | 2.6 | -1.525177 |
| 47 | 73 | | Male | 6.5 | 71.4 | 3.4 | 1.525177 |
| 48 | 77 | | Male | 1 | 14.8 | 10.6 | -2.643439 |
| 49 | 70 | | Male | 1.8 | 2328 | 7.3 | 1.525177 |
| 50 | 55 | | Male | 14.9 | 507 | 11.6 | 1.525177 |
| 51 | 55 | | Male | 16.9 | 26 | 10 | 1.525177 |
| 52 | 65 | | Male | 9.4 | 774 | 6.9 | 1.525177 |
| 53 | 69 | | Male | 29.8 | 8.8 | 8.2 | -5.693793 |
| 54 | 68 | | Famale | 8 | 8300 | 13.6 | 1.525177 |
| 55 | 66 | | Male | 27.9 | 25 | 7.7 | -1.525177 |
| 56 | 66 | | Male | 23.9 | 15.7 | 16.5 | -8.857136 |
| 57 | 66 | | Male | 9.7 | 3660 | 11.3 | 1.525177 |
| 58 | 61 | | Male | 7.6 | 8.4 | 4.4 | -2.643439 |
| 59 | 63 | | Male | 23.7 | 17 | 6.6 | -1.525177 |
| 60 | 53 | | Male | 6.7 | 159 | 10.6 | 1.525177 |
| 61 | 51 | | Famale | 6.4 | 61.4 | 6.8 | 1.525177 |
| 62 | 55 | | Male | 44.9 | 58.9 | 8.9 | -1.525177 |
| **Validation set** | |  |  |  |  |  |  |
| 63 | 55 | | Male | 42.7 | 8.9 | 18.4 | -8.857136 |
| 64 | 57 | | Male | 12.6 | 4.2 | 24.1 | -5.806782 |
| 65 | 58 | | Male | 11.5 | 5 | 10 | -2.643439 |
| 66 | 63 | | Male | 3.7 | 12.2 | 5.1 | -2.643439 |
| 67 | 51 | | Male | 10.5 | 255 | 7.6 | 1.525177 |
| 68 | 52 | | Male | 32.7 | 8.8 | 70.6 | -8.857136 |
| 69 | 62 | | Male | 28 | 30.7 | 26.7 | -4.68852 |
| 70 | 58 | | Male | 25.4 | 23.3 | 35.1 | -4.68852 |
| 71 | 64 | | Male | 42.5 | 4.3 | 20.8 | -8.857136 |
| 72 | 65 | | Male | 7.6 | 14.9 | 11.6 | -2.643439 |
| 73 | 61 | | Male | 20.8 | 6.4 | 4 | -5.693793 |
| 74 | 55 | | Male | 22.3 | 6.8 | 13.5 | -5.693793 |
| 75 | 56 | | Male | 24.6 | 12.7 | 25.7 | -8.857136 |
| 76 | 62 | | Male | 20.6 | 80 | 45 | -4.68852 |
| 77 | 64 | | Famale | 7.4 | 10.7 | 10.9 | -2.643439 |
| 78 | 55 | | Famale | 35.8 | 6.8 | 26.1 | -8.857136 |
| 79 | 72 | | Male | 17.3 | 6.4 | 62.1 | -5.806782 |
| 80 | 67 | | Male | 17.9 | 3.1 | 8.8 | -5.693793 |
| 81 | 67 | | Famale | 15 | 38 | 8.9 | 1.525177 |
| 82 | 71 | | Male | 23.5 | 13.4 | 24.6 | -8.857136 |
| 83 | 72 | | Famale | 16 | 2.2 | 31.7 | -5.806782 |
| 84 | 68 | | Famale | 32.8 | 3.2 | 18.5 | -8.857136 |
| 85 | 67 | | Famale | 24.2 | 3.7 | 35.8 | -8.857136 |
| 86 | 59 | | Male | 18.8 | 5.1 | 64.1 | -8.857136 |
| 87 | 62 | | Male | 44.1 | 1.84 | 63.8 | -8.857136 |
| 88 | 65 | | Male | 12 | 27.4 | 7.8 | 1.525177 |
| 89 | 52 | | Male | 28.4 | 13.5 | 92.7 | -8.857136 |
| 90 | 53 | | Male | 54.6 | 8.9 | 19 | -8.857136 |
| 91 | 66 | | Male | 18.2 | 14.5 | 13.7 | -5.693793 |
| 92 | 69 | | Male | 10.1 | 202 | 7.5 | 1.525177 |
| 93 | 67 | | Male | 6.5 | 205 | 3.2 | 1.525177 |
| 94 | 68 | | Male | 8.4 | 50 | 1.7 | 1.525177 |
| 95 | 60 | | Male | 7.7 | 19.8 | 7.2 | 1.525177 |
| 96 | 49 | | Male | 10.1 | 37 | 7.7 | 1.525177 |
| 97 | 51 | | Male | 16.4 | 13 | 21.7 | -5.806782 |
| 98 | 65 | | Famale | 50.5 | 5.1 | 24.2 | -8.857136 |
| 99 | 65 | | Male | 10.2 | 10.7 | 3.9 | -2.643439 |
| 100 | 63 | | Male | 6.1 | 613 | 6.8 | 1.525177 |
| 101 | 47 | | Male | 11 | 20.4 | 5.7 | 1.525177 |
| 102 | 54 | | Famale | 12.2 | 4.8 | 7.1 | -2.643439 |
| 103 | 67 | | Male | 12.2 | 18 | 1.4 | 1.525177 |
| 104 | 67 | | Male | 14.5 | 8.6 | 96.9 | -5.806782 |
| 105 | 65 | | Male | 11.2 | 16.9 | 15.7 | -1.638166 |
| 106 | 65 | | Male | 15.1 | 47 | 8.7 | 1.525177 |
| **Independent**  **validation set** | | | |  |  |  |  |
| 107 | 55 | | Male | 5 | 43.8 | 1.9 | 1.525177 |
| 108 | 55 | | Male | 21 | 41.7 | 23.1 | -4.68852 |
| 109 | 49 | | Male | 6.6 | 38.8 | 10.2 | 1.525177 |
| 110 | 64 | | Male | 15.2 | 41.9 | 6.3 | 1.525177 |
| 111 | 68 | | Male | 13.2 | 48.4 | 5.3 | 1.525177 |
| 112 | 69 | | Famale | 13.1 | 38.9 | 8.9 | 1.525177 |
| 113 | 52 | | Famale | 18.7 | 35.6 | 12.2 | -1.525177 |
| 114 | 42 | | Male | 23.3 | 77.1 | 14.1 | -4.68852 |
| 115 | 58 | | Male | 46.1 | 55.7 | 17.3 | -4.68852 |
| 116 | 59 | | Male | 4 | 22.9 | 24.6 | -1.638166 |
| 117 | 70 | | Male | 15.9 | 34.4 | 18.8 | -1.638166 |
| 118 | 51 | | Male | 7.4 | 43.4 | 16 | -1.638166 |
| 119 | 62 | | Male | 11.9 | 29.1 | 14 | -1.638166 |
| 120 | 36 | | Male | 10.1 | 16.5 | 8.1 | -2.643439 |
| 121 | 61 | | Male | 20.4 | 47.6 | 7.2 | -1.525177 |
| 122 | 55 | | Male | 8.4 | 51.9 | 0 | 1.525177 |
| 123 | 55 | | Male | 17.5 | 45.1 | 12.1 | 1.525177 |
| 124 | 63 | | Male | 11.8 | 40.7 | 27.4 | -1.638166 |
| 125 | 71 | | Famale | 8 | 33.3 | 7.3 | 1.525177 |
| 126 | 60 | | Famale | 18.2 | 50.1 | 10.4 | -1.525177 |
| 127 | 61 | | Male | 21.8 | 53.2 | 10.2 | -1.525177 |
| 128 | 63 | | Male | 6.7 | 48.1 | 2.5 | 1.525177 |
| 129 | 67 | | Male | 10.5 | 36.8 | 20.1 | -1.638166 |
| 130 | 72 | | Male | 6.1 | 30.9 | 5.9 | 1.525177 |
| 131 | 76 | | Male | 10.4 | 32.7 | 7.9 | 1.525177 |
| 132 | 59 | | Male | 8.1 | 29.3 | 9 | 1.525177 |

**Table S9. Sequences of the primers used for qRT-PCR.**

| Bacteria | Primer sequence (5’→3’) | Amplification size (bp) |
| --- | --- | --- |
| *TMx* | F: TCGCTTCGAATGGGGTATGG R: AGCTCAGCCTTCGCTTTGAT | 84 |
| *Prevotella* | F: CCTACTGATCGTCGCCTTGG  R: GTAACGCGTATCCAACCTGC | 177 |
| *Sphingobacterium* | F: GAGCCCCTAGACATCGTAGC  R: TGAGCAACCTGCCCATATCAG | 174 |
